# Range expansion and first observation of *Tridacna noae* (Cardiidae: Tridacninae) in American Sāmoa

**DOI:** 10.1002/ece3.9635

**Published:** 2022-12-28

**Authors:** Paolo Marra‐Biggs, James Fatherree, Alison Green, Robert J. Toonen

**Affiliations:** ^1^ Hawai'i Institute of Marine Biology University of Hawai'i at Mānoa Kāne'ohe Hawaii USA; ^2^ Hillsborough Community College Tampa Florida USA; ^3^ Alison Green Marine Gold Coast Queensland Australia

**Keywords:** conservation, giant clams, mitogenome, Tridacninae

## Abstract

Giant clams are ecologically important, benefitting species of all trophic levels. But numerous populations have declined drastically in numbers due to past intensive exploitation that led to their listing in both CITES Appendix II and IUCN Red List of Threatened Species. However, giant clams are notoriously difficult to identify, and recent molecular work has revealed that morphological misidentification of giant clams have confounded current population assessments and extinction risk. The most recent study of the status of giant clams in the Samoan Archipelago was published in a journal over 20 years ago, without molecular corroboration of visual identifications. Using morphologic characteristics and ezRAD genetic techniques, we identify the existence of *Tridacna noae* in the Samoan Archipelago, presenting the first observation and a resulting range expansion. Accurately identifying the extant species in the archipelago is the first step toward a much‐needed population status assessment to effectively manage these long‐lived species.

## Graphical abstract

Using morphologic characteristics and ez‐RAD genetic techniques, we identify the existence of Tridanca noae in the Samoan archipelago, presenting the first observation and a resulting range expansion. This is the first step in a much‐needed comprehensive assessment of population stocks in the archipelago.

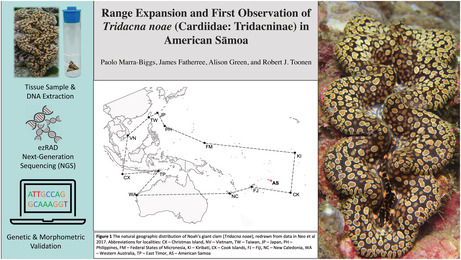

Giant clams of the subfamily Tridacninae are conspicuous members of Pacific coral reef communities that play such important ecological roles that some authors have considered them as keystone species (Guibert et al., 2020; Mamat et al., 2021). All species of Tridacninae are of widespread conservation concern and are listed on both the Convention on International Trade in Endangered Species of Wild Fauna and Flora (CITES) and the International Union for Conservation of Nature (IUCN). However, giant clams are notoriously difficult to identify, and recent molecular work has revealed that morphological misidentification of giant clams have confounded current population assessments and extinction risk (Neo et al., 2017; Tisdell, 1989; Wells, 1997). As a case in point, *Tridacna noae* that was first described by C. Röding in 1798 became synonymized to *T. maxima* as it appeared to be a variant of the latter species based on shell morphology (Rosewater, 1965). Only recently has *T. noae* been resurrected as a valid species distinct from its congener *T. maxima* based on both morphological and molecular data (Su et al., 2014).

Neo et al. (2017) present the most comprehensive survey of giant clam species distributions and status, but no samples from the Samoan archipelago were included in that study. In previous studies by Fauvelot et al., 2019, a team sampled Upolu, Independent Sāmoa finding individuals belonging to *T. noae*. However, the most recent published studies conducted on tridacnine clams in the territory of American Sāmoa were published nearly 20 years ago (Green & Craig, 1999). This study was conducted prior to recent genetic recognition of *T. noae* (Su et al., 2014), so the authors assumed all visually similar samples were *T. maxima* (A. Green, pers. obs.), which would confound population density estimates if *T. noae* were also present in American Sāmoa. As genetic techniques are more commonly used to confirm species, more cases of *T. noae* are being identified, as seen in Militz et al. (2015).

Previously, American Sāmoa hosted two species of giant clams in the territorial waters: *Tridacna squamosa* and *T. maxima*. Fossilized shells indicate a third species, *Hippopus hippopus*, used to occur in Sāmoa, but is now locally extinct (Nagaoka, 1993; Newman & Gomez, 2000). Additionally, an aquaculture program was started for juvenile *H. hippopus*, *T. gigas*, and *T. derasa*, but stocks were harvested prior to reproduction and appear to be functionally extirpated. Here, we use next‐generation sequencing to confirm identification of giant clams and expand the species range of *T. noae* to include American Sāmoa (Figure 1). Additionally, this discovery highlights the need to revisit historical population assessments to determine the distribution and abundance of each *T. noae* and *T. maxima* across the Samoan Archipelago.

**FIGURE 1:**
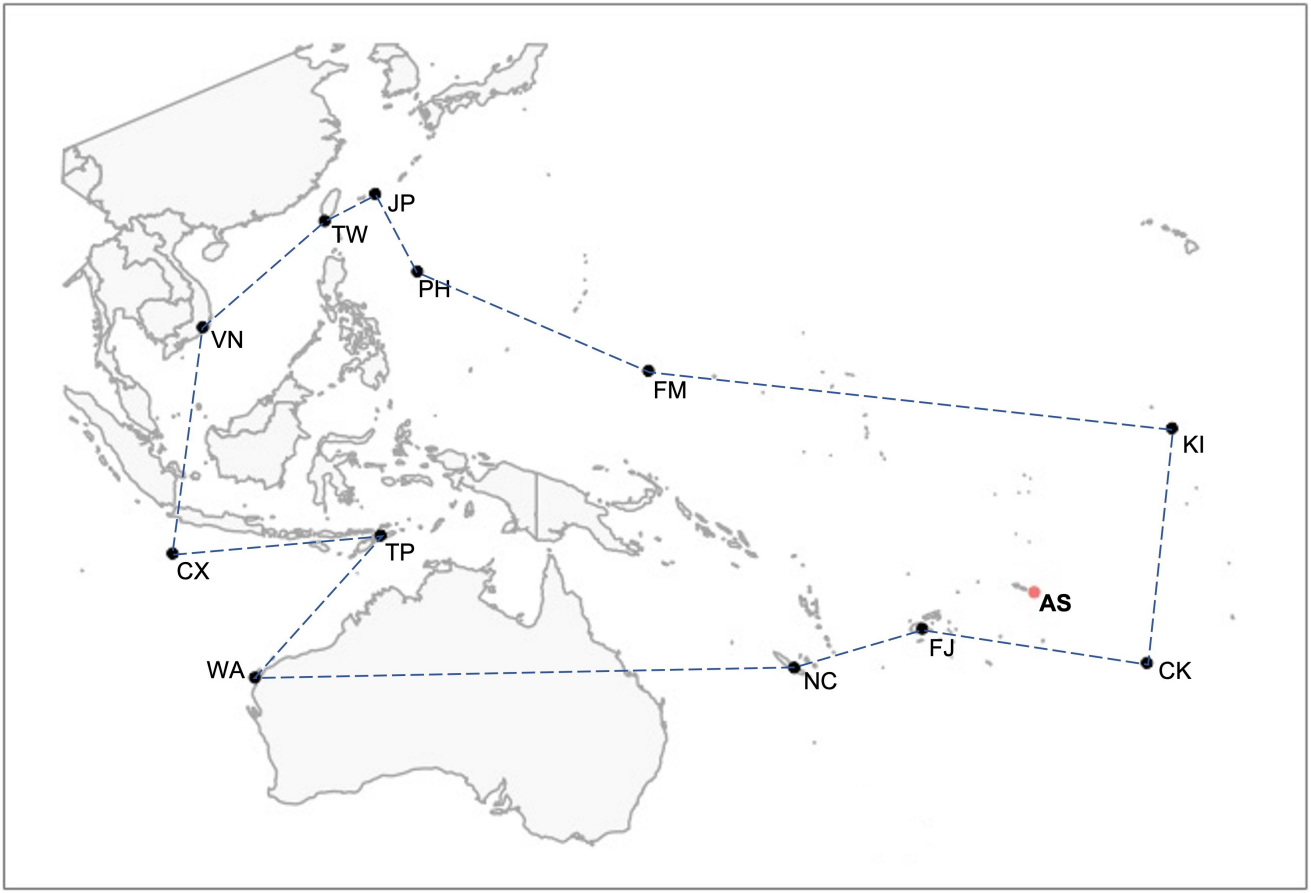
The natural geographic distribution of Noah's giant clam (*Tridacna noae*), redrawn from data in Neo et al., 2017. Abbreviations for localities: AS, American Samoa; CK, Cook Islands; CX, Christmas Island; FJ, Fiji; FM, Federal States of Micronesia; JP, Japan; KI, Kiribati; NC, New Caledonia; NV, Vietnam; PH, Philippines; TP, East Timor; TW, Taiwan; WA, Western Australia.

Tissue biopsies were collected from four clams, two putative *T. noae* and two putative *T. maxima*, found during surveys on Tutuila, American Sāmoa, with in situ photos taken of each individual sampled, seen in Figure 2. Clams were sampled from depths between 27 and 37 ft and ranged from 12 to 20 cm in antero‐posterior shell length. Clams were identified morphologically as either *T. noae* or *T. maxima* (following Fatherree, 2016; Militz et al., 2015) and then sequenced using reduced representation genomic sequencing (ezRAD, Toonen et al., 2013). Reads were mapped to reference mitogenomes from Tan et al. (2022) using Minimap2 (Li, 2018) in Geneious Prime v2021.1.1 (www.geneious.com).

**FIGURE 2:**
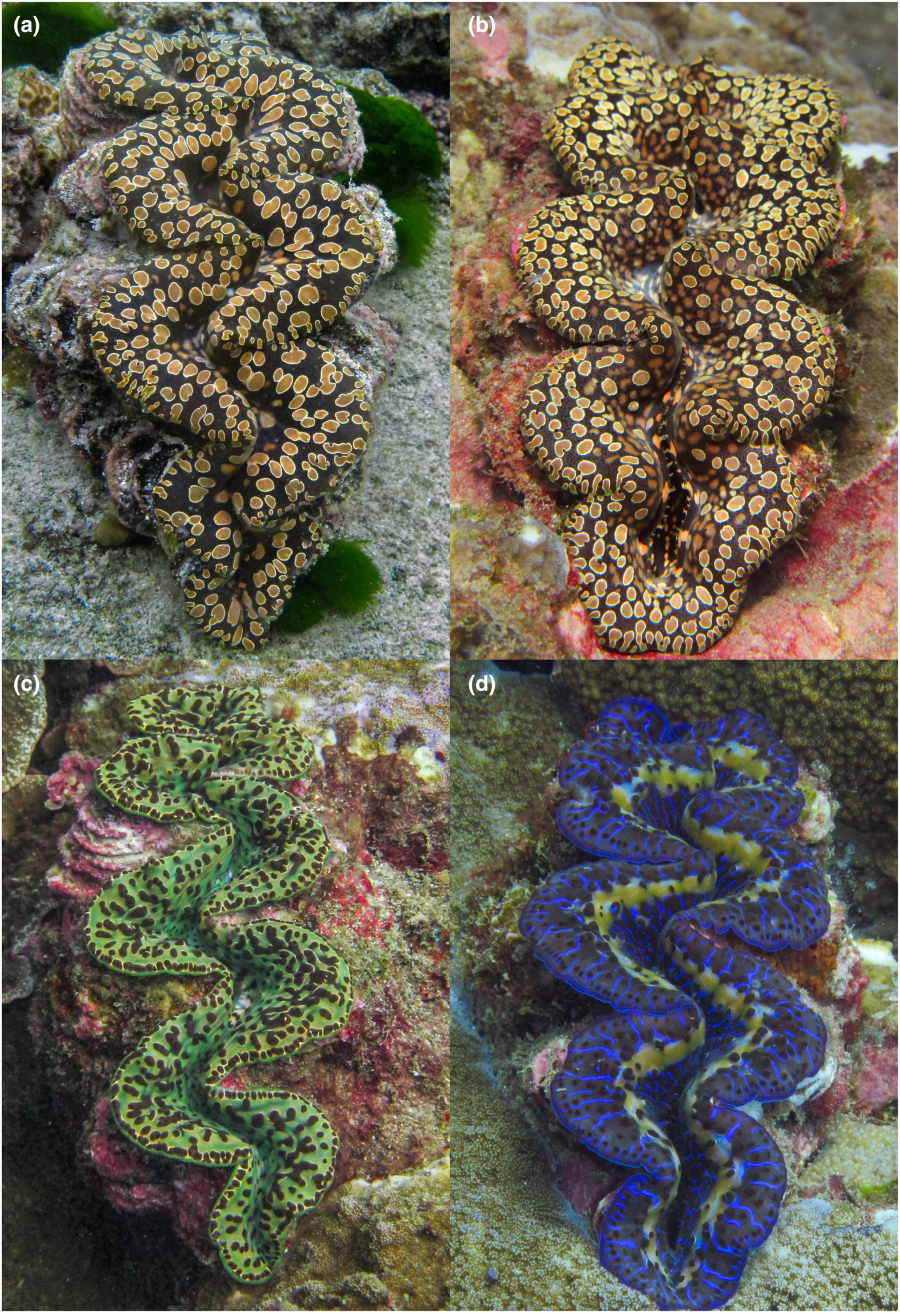
*Tridacna noae* (a, b) from Tutuila, American Sāmoa, showing the teardrop‐shaped spots on the edge of the mantle with the typical golden ring, and the lack of a distinct row of eyes (or hyaline organs). *Tridacna maxima* (c, d) shows considerable variation in the color on the mantle, but a row of eyes along the periphery of the mantle is a distinctive character. (c) *T. maxima* and (d) a blue variant of *T. maxima* from Tutuila, American Sāmoa for comparison highlighting the row of eyes at the upper mantle's margin and lacking the ringed spots. Photos: Paolo Marra‐Biggs.

The two individuals identified morphologically as *T. noae* (Figure 2) were confirmed genetically with nearly complete mitogenomes 15,240 ± 4 base pairs in length (86.5 ± 1.2%), and with high coverage depth (106.2 ± 75×), that matched the Tan et al. (2022) reference sequence provided by Danwei Huang at 99.7 ± 0.05% pairwise identity. Another individual identified morphologically as *T. maxima* was also confirmed genetically with a nearly complete mitogenome of 14,978 base pairs (80.7%), with relatively high coverage depth (53.5×), matching at 99.6% pairwise identity to the *T. maxima* reference sequence (Tan et al., 2022). Given this clear distinction among the morphologically identified samples, the final *T. maxima* sample was not sequenced here.

There is considerable morphological variation within American Samoan *T. maxima* (Figure 2c,d) and *T. noae*. Physical characteristics that are typically used for identification (such as the size of byssal orifice, number of radial folds in the rib interstices, elaborate vs. simple incurrent siphon tentacles, size of scutes) are variable within the species, which can lead to confusion or misidentification between *T. maxima* and *T. noae*. Additionally, the mantle can vary dramatically in color, pattern, and texture and are unreliable as proxies for species identification (Su et al., 2014). As with the many phenotypic morphs of *Tridacna maxima*, *T. noae* can exhibit two color morphs, brown and blue (Militz et al., 2015).

Many of these characteristics also fluctuate based on individual health (Mies, 2019). However, certain morphologic characteristics can aid in distinguishing between *T. maxima* and *T. noae*. Examples of features more commonly observed in *T. noae* are “tear‐drop” oval patterns on the mantle edge and less pronounced hyaline organs (Su et al., 2014).

Based on these findings, we can update the catalog of extant species in American Sāmoa to include: *Tridacna maxima*, *Tridacna squamosa*, and now *Tridacna noae*. By confirming the identification of these two sometimes difficult to distinguish clam species genetically along with photo‐documentation to highlight morphological distinctions, we hope to improve the efficacy and accuracy of identification of *T. noae* and *T. maxima* during future field surveys. Tridacnid clams are listed as a priority species under the territory's Local Action Strategy, and accurately identifying species is critical to understanding the distribution and abundance of each and can help ensure the appropriate and most effective management of these long‐lived species.

## AUTHOR CONTRIBUTIONS

**Paolo Giannino Marra‐Biggs:** Conceptualization (equal); data curation (lead); formal analysis (lead); funding acquisition (equal); investigation (equal); methodology (lead); project administration (equal); resources (equal); software (lead); supervision (equal); validation (lead); visualization (equal); writing – original draft (lead); writing – review and editing (lead). **James Fatherree:** Data curation (supporting); investigation (supporting); resources (supporting); writing – review and editing (supporting). **Alison Green:** Conceptualization (supporting); methodology (supporting); resources (supporting); writing – review and editing (supporting). **Rob Toonen:** Conceptualization (equal); data curation (supporting); formal analysis (supporting); funding acquisition (equal); investigation (equal); methodology (supporting); project administration (equal); resources (equal); software (supporting); supervision (equal); validation (supporting); visualization (equal); writing – original draft (supporting); writing – review and editing (equal).

### OPEN RESEARCH BADGES

This article has earned Open Data and Open Materials badges. Data and materials are available at https://www.ncbi.nlm.nih.gov/sra/PRJNA867692.

## COMPLIANCE

Sampling of giant clams was conducted under guidance of the National Park of American Sāmoa and under the scientific permit of American Samoan Government: Department of Marine and Wildlife, permit no. DMWR‐2016/046.

## Data Availability

Upon publication, the data that support the findings of this study are openly available in “NCBI Short Read Archive” at https://www.ncbi.nlm.nih.gov/sra/PRJNA867692, with a BioProject number [PRJNA867692], and sample reference numbers [SAMN30219961, SAMN30219962, SAMN30219963].
